# Together we stand, resilient we stay : The effect of minority stress and resilience on transgender mental health

**DOI:** 10.1192/j.eurpsy.2021.1620

**Published:** 2021-08-13

**Authors:** A.B. Sahin, D. Buyukgok

**Affiliations:** 1 Department Of Psychiatry, Basaksehir Cam and Sakura City Hospital, Istanbul, Turkey; 2 Department Of Psychiatry, Istanbul University Istanbul Faculty of Medicine, Istanbul, Turkey

**Keywords:** Transgender, resilience, minority stress, mental health

## Abstract

**Introduction:**

Prejudice, stigmatization and discrimination behaviors causes social stress and lead vulnerability to mental and physical health problems in Transgender and Gender Nonconforming (TGNC) individuals. The prevalence of mental disorders that can be associated with “minority group stress”, especially major depression and anxiety disorders, are known to be higher in the TGNC group in comparison to general population.

**Objectives:**

The aim of this study was to reveal the impact of minority stress on TGNC individuals’ mental health. Resilience factors like gender identity pride, social support, community connectedness expected to diminish the negative impact of stigmatization and discrimination.

**Methods:**

The study sample consisted of 48 volunteered participants who consulted to Psychiatry Department for gender transition process. After semi-structured interview, applicants were given Gender Minority Stress and Resilience Scale-Turkish Form (GMSR-Tr), Beck Depression Inventory (BDI), Beck Anxiety Inventory (BAI), Perceived Stress Scale (PSS), Multidimensional Scale of Perceived Social Support (MSPSS).

**Results:**

Analysis revealed a negative correlation between the stress subscales of GMSR-Tr scale and BDI (p< .001; r_s_= .727), BAI (p< .001; r_s_= .649), PSS (p< .001; r_s_= .671). For psychological resilience, the strongest positive relationship was found with the community connectedness subscale (p< .001; r_s_= .864); the strongest negative relationship was observed with the internalized transphobia subscale (p< .001; r_s_= .750).

**Conclusions:**

Our study presented the importance of internalized transphobia and protective effect of resilience factors for mental health outcomes of TGNC individuals exposed to minority stress. The depression, anxiety and stress scores decreases with increasing psychological resilience.

